# Pregnant women carrying female fetuses are at higher risk of placental malaria infection

**DOI:** 10.1371/journal.pone.0182394

**Published:** 2017-07-28

**Authors:** Ishag Adam, Magdi M. Salih, Ahmed A. Mohmmed, Duria A. Rayis, Mustafa I. Elbashir

**Affiliations:** 1 Faculty of Medicine University of Khartoum, Sudan; 2 Faculty of Medical Laboratory Sciences, University of Khartoum, Sudan; 3 Faculty of Medicine, Ribat University, Sudan; Universidade de Sao Paulo Instituto de Ciencias Biomedicas, BRAZIL

## Abstract

**Background:**

The pathophysiology of the placental malaria is not fully understood. If there is a fetal sex–specific susceptibility to malaria infection, this might add to the previous knowledge on the immunology, endocrinology and pathophysiology of placental malaria infections.

**Aims:**

This study was conducted to assess whether the sex of the fetus was associated with placental malaria infections.

**Subjects and methods:**

A cross-sectional study was performed including a secondary analysis of a cohort of women who were investigated for prevalence and risk factors (including fetal sex) for placental malaria in eastern Sudan. Placental histology was used to diagnose placental malaria infections.

**Results:**

Among 339 women enrolled, the mean (SD) age was 25.8 (6.7) years and parity was 2.7 (2.2). Among the new born babies, 157 (46.3%) were male and 182 (53.7%) were female. Five (1.5%), 9 (2.7%) and 103 (30.4%) of the 339 placentas had active, active-chronic, past-chronic malaria infection on histopathology examination respectively, while 222 (65.5%) of them showed no malaria infection. Logistic regression analyses showed no associations between maternal age or parity and placental malaria infections. Women who have blood group O (OR = 1.95, 95% CI = 1.19–3.10; *P* = 0.007) and women who had female new born were at higher risk for placental malaria infections (OR = 2.55, 95% CI = 1.57–4.13; *P<* 0.001).

**Conclusion:**

Fetal gender may be a novel risk factor for placental malaria. In this work the female placentas were at higher risk for malaria infections than the male placentas.

## Introduction

There is a great in interest in recent years in the female/male distribution during pregnancy and its interaction with maternal and perinatal health [1]. Many maternal physiological and endocrine functions are influenced in a fetal sex-specific pattern during pregnancy [2]. Various disparities between male and female fetuses in relation to a number of adverse pregnancy outcomes have been recently observed e.g. stillbirth, preeclampsia and fetal growth restriction [3–8].

*Plasmodium falciparum* malaria infection during pregnancy is a major public health problem especially in sub-Saharan Africa, where it is a major cause of maternal and perinatal morbidity and mortality [9,10]. The mechanisms leading to morbidity and mortality in placental malaria are incompletely understood. However, an inflammatory response in the placenta with accumulation of monocytes and macrophages in the maternal vascular bed of the placenta has been related to both severe anemia in the mother and low birth weight in the new born [11–13]. The pathophysiology of the placental malaria is not fully understood and the finding of the fetal sex–specific susceptibility of malaria infection might add to the previous knowledge on the immunology, endocrinology and pathophysiology of placental malaria infections. Pregnant women in Sudan are at higher risk for malaria and malaria during pregnancy has been reported as main cause of maternal and perinatal adverse effect [10,14]. This study was conducted to assess whether the sex of the fetus was associated with placental malaria infections where there is no published data on fetal sex and placental malaria infection.

## Material and methods

This is a secondary analysis of a cohort of women who were investigated for prevalence and risk factors for placental malaria in eastern Sudan using placental histology as tool for diagnosis for placental malaria infections [15,16]. The details of the methods have been mentioned before. In summary, parturient women who delivered a singleton baby in New Half (October 2006–March 2007) and Gadarif (November 2007–January 2008) hospitals in eastern Sudan were approached to participate in the study. After signing a witnessed informed consent, socio-demographic, medical and obstetrics characteristics were gathered by questionnaires that were completed by trained medical officers in the local Arabic language. Women with antepartum haemorrhage or thyroid disease were excluded from the study. Body mass index (BMI) was computed from the woman’s weight and height (weight in kg/ height in m^2^ that were taken and recoded immediately. Following delivery, new born sex and weight were recorded.

### Hematology

Thick blood films for malaria were prepared from the maternal, placental (small piece (0.5 cm3) excised from the centre) and cord blood and were Giemsa-stained and the number of asexual *P*. *falciparum* parasites per 200 white blood cells was recorded. Maternal blood groups were investigated by the agglutination method.

### Histopathology

Around three cm^3^ full thickness placental blocks were taken from the placenta and kept in neutral buffered formalin for histopathology examinations. The biopsy samples were processed by embedding them in paraffin wax using standard techniques. In every case, the thick paraffin sections were stained with hematoxylin-eosin and Giemsa stains. Histology was evaluated using the criteria of Bulmer *et al* [17]. Placentas were identified as not infected, no evidence of parasite or pigment; active infection, parasites in maternal erythrocytes in the intervillous space, pigment in erythrocytes and monocytes in the intervillous space but no pigment in fibrin or cells within fibrin; active-chronic infection, parasites in maternal erythrocytes in the intervillous space, pigment in erythrocytes and circulating monocytes within the intervillous space and pigment in fibrin or cells within fibrin and/or chorionic villous syncytiotrophoblast or stroma; or past-chronic infection, parasites not present, pigment confined to fibrin or cells within fibrin.

The histopathology and hematology studies were performed by two independent readers namely HAH for hematology and AAM for histopathology.

### Statistics

Data were entered using SPSS for windows (version 20.0). Means and proportions were compared between two groups (according to placental malaria infection and male/female new born) using Student’s t-test and X^2^ tests, respectively. Binary regressions were performed with placental malaria infections as a dependent variable and the independent variables age, parity, residence, education, antenatal care, maternal blood group (O versus non O) and new-born gender were entered in the model if their univariate P was <0.20. Odds ratios and 95% confidence interval were calculated and *P* < 0.05 was considered significant.

### Ethics

The study received ethical clearance from the Research Board at the Faculty of Medicine, University of Khartoum.

## Results

### General characteristics

Thirty –two women were excluded from the study because they had incomplete data (18), had antepartum hemorrhage (11) or had thyroid diseases (3). Three hundred and thirty nine women had complete data include the placental malaria infections and the sex of the new born and their data were analysed. Of these women, 139 (41.0%), 265 (78.2%), 113 (33.3%) and 66 (19.55%) women were primiparae, had rural residence, were illiterate and had no antenatal care, respectively. While 32.2% of these women used bed nets, only (5.3%) used intermittent preventive treatment in the index pregnancy. The mean (SD) of the age of the enrolled women was 25.8 (6.7) years and mean (SD) parity was 2.7 (2.2), respectively. Of the participants, 79 (23.3%), 74 (21.8%), 22 (6.5%) and 164 (48.4%) had blood group A, B, AB and O, respectively.

One hundred and fifty seven (46.3%) of the new born babies were males and 182 (53.7%) were females. The mean (SD) of the birth weight was 3081.4 (530) g and it was not different between males and females [3122.7 (526.0) vs. 3045.8 (534) g, P = 0.184. There was no significant difference in the maternal age, parity, education, residence, antenatal care level, or proportion of women using bed nets in the index pregnancy between women with male and female new born babies, Table 1.

**Table 1:** Comparing basic characteristics between women with male and women with female new born.

Variables	Male new born	Female new born	P
	(n = 157)	(n = 182)	
*Mean (SD) of*			
Age, years	26.1 (6.8)	25.5 (6.6)	0.518
Parity	2.8 (2.2)	2.6 (2.1)	0.321
Body mass index, kg/m^2^	23.2 (3.7)	23.1 (3.3)	0.845
Birth weight, g	3122.7 (526.0)	3045.8 (534.0)	0.184
*Number (%) of*			
Rural residence	123 (78.3)	142 (78.0)	0.568
Illiterate	54 (34.4)	59 (32.4)	0.570
Lack of antenatal care	35 (22.3)	31 (17.0)	0.223
Used bet nets	48 (30.6)	61 (33.5)	0.641
Intermittent preventive treatment	10 (6.4)	8 (4.4)	0.472
Blood group O	85 (54.1)	79 (43.4)	0.062

### Malaria

Maternal and placental blood films for malaria were positive in three cases. Two maternal, placental and cord settings had positive blood films for malaria. All the positive blood films for malaria were found to have a positive placental malaria infection on histology.

One hundred and seventeen placentas had malaria infection. Five (1.5%), 9 (2.7%) and 103 (30.4%) of the 339 placentas had active, active-chronic, past-chronic malaria infection on histopathology examination respectively, while 222 (65.5%) of them showed no malaria infection.

While there was no significant difference in age, parity, education, residence and antenatal care level between women with placental malaria infection and women without placental malaria infection, women with placental malaria infection had a significantly higher number of O blood group and had a female new born (78 (66.7%) vs104 (46.8%), P < 0.001), Table 2.

**Table 2:** Comparing basic characteristics between women with placental malaria infection and the women without malaria infection.

Variables	Placental malaria	No placental malaria	P
	infection (n = 117)	infection (n = 222)	
*Mean (SD) of*			
Age, years	26.4 (6.5)	25.5 (6.8)	0.211
Parity	2.9 (2.1)	2.6 (2.2)	0.172
Body mass index, kg/m^2^	23.5 (4.0)	23.0 (3.2)	0.255
*Number (%) of*			
Rural residence	90 (76.9)	175 (78.8)	0.681
Illiterate	38 (32.5)	75 (33.8)	0.901
Lack of antenatal care	20 (17.1)	46 (20.7)	0.472
Used bet nets	36 (30.8)	73 (32.9)	0.349
Intermittent preventive treatment	6 (5.1)	12 (5.4)	0.986
Blood group O	66 (56.4)	98 (44.1)	0.032
Female gender of the new born	78 (66.7)	104(46.8)	< 0.001

Logistic regression analyses showed no associations between the age, parity, education, antenatal care and placental malaria infections. Women who have blood group O (OR = 1.95, 95% CI = 1.19–3.10; *P* = 0.007) and women who had female new born were at higher risk for placental malaria infections (OR = 2.55, 95% CI = 1.57–4.13; *P<* 0.001), Table 3.

**Table 3:** Binary logistic regression analyses of the factors associated with placental malaria infection in eastern Sudan.

Variables	Odd ratio	95% Confidence interval	P
Age, years	1.02	0.97−1.06	0.356
Parity	1.05	0.92 −1.19	0.446
Blood group O	1.92	1.19 − 3.10	0.007
Female gender of the new born	2.55	1.57 − 4.13	< 0.001

## Discussion

The study showed that age and parity were not associated with placental malaria infection. This agrees with the findings of the parent studies which have been discussed in detail elsewhere [15,16].

Interestingly the current study showed that women who were pregnant with females were at 2.55 at higher risk for placental malaria infection. To the best of our knowledge this is the first study to investigate the relationship between fetal sex and the susceptibility to malaria infection. Recently Laar *et al*., observed that female infants of HIV infected women had an increased prevalence of cord blood malaria parasitemia [18]. On the other hand, it has previously been reported that male sex was independently associated with a baby being born with umbilical cord blood parasitemia [19]. Griffin et al. reported no significant difference between male and female sex and maternal *P*. *falciparum* parasitaemia in the first half of pregnancy.

A time may come that studies of this kind and similar cohorts or re-analyzing the previous data on malaria infection during pregnancy in different settings may conform the current observation of an association between fetal sex and susceptibility to placental malaria. If the future research yields the same results, then the expected hypothesis would be how does a female fetus render a woman more susceptible to malaria infection? The attractiveness to the mosquitoes, the hormones (cortisol and prolactin), immune system modulation and increase in the receptors mediating parasite cytoadhesion need to investigated as possible mechanisms of association between female sex and placental malaria infection [20,21]. One cellular mechanism involving glucocorticoid receptor isoforms has been identified and could explain the difference between a female versus male placenta in the response to a maternal exposure to cortisol and hence malaria infections [22].

Although the effects of fetal sex on the long-term health of the mother are still not completely understood, these are now obvious in two maternal diseases namely asthma and preeclampsia [23,24]. It is worth mentioning that, in general, male fetuses and placentas are postulated to be more susceptible to adversity in utero[25].

The placenta not only transports nutrients and oxygen from the maternal circulation and returns waste products to the maternal circulation, but also it has a major endocrine and immunological function being responsible for synthesizing many hormones and cytokines that might have effects on the physiology of both the mother and the fetus [26,27]. These hormones and cytokines, such as soluble endoglin, and insulin-like growth factor, acid labile subunit, leptin and C-peptide, might have their sex-specific characteristics [28,29]. Unfortunately, the placenta has inherently been considered an asexual organ and the sex of the embryo was not considered in research on placenta functions [30]. But it is now obvious that the placenta has a sex of the embryo it belongs to [30,31].

The pathophysiology of placental malaria is not fully understood and the finding of the sex–specific susceptibility will add to the previous knowledge on the immunology, endocrinology and pathophysiology of placental malaria infections.

### Limitation of the study

The submicroscopic malaria was not investigated (through polymerase chain reaction) and this point should be considered as one of the limitation of the current study. Previous study has shown that 32.0% of pregnant women in eastern Sudan had submicroscopic malaria [32]. Many factors e.g. maternal and paternal factors that could influence fetal sex were not investigated [33]. The data that were re-analyzed in the current study were data almost 10 years back. Perhaps there are some changes in the epidemiology and transmission of malaria in the area e.g. emergence of *P*. *vivax* in the area [34].

### Conclusion

The current study supported the recent findings that fetal sex might influence the maternal milieu. In this work the female placentas were at higher risk for malaria infections than the male placentas.

### Ethics

The study received ethical clearance from the Research Board at the Faculty of Medicine, University of Khartoum, Sudan.

## Supporting information

S1 Table Raw data. (XLSX)
